# The importance of migratory drop-off for island colonization in birds

**DOI:** 10.1098/rspb.2023.2926

**Published:** 2024-04-17

**Authors:** Paul Dufour, Ferran Sayol, Rob Cooke, Tim M. Blackburn, Laure Gallien, Michael Griesser, Manuel J. Steinbauer, Søren Faurby

**Affiliations:** ^1^ Department of Biological & Environmental Sciences, University of Gothenburg, Gothenburg, Sweden; ^2^ Gothenburg Global Biodiversity Centre, Gothenburg, Sweden; ^3^ Centre for Ecological Research and Forestry Applications (CREAF), E08193 Bellaterra (Cerdanyola del Vallès), Catalonia, Spain; ^4^ UK Centre for Ecology & Hydrology, Maclean Building, Crowmarsh Gifford, Wallingford, Oxfordshire OX10 8BB, UK; ^5^ Centre for Biodiversity and Environment Research, Department of Genetics, Evolution and Environment, University College London, London WC1E 6BT, UK; ^6^ Institute of Zoology, Zoological Society of London, London NW1 4RY, UK; ^7^ LECA, CNRS, Univ. Grenoble Alpes, Univ. Savoie Mont Blanc, Chambéry, France; ^8^ Department of Biology, University of Konstanz, Konstanz, Germany; ^9^ Center for the Advanced Study of Collective Behavior, University of Konstanz, Konstanz, Germany; ^10^ Department of Collective Behavior, Max Planck Institute of Animal Behavior, Konstanz, Germany; ^11^ Bayreuth Center of Ecology and Environmental Research (BayCEER) & Bayreuth Center of Sport Science (BaySpo), University of Bayreuth, Bayreuth, Germany; ^12^ Department of Biological Sciences, University of Bergen, Bergen, Norway

**Keywords:** seasonal migration, long-distance dispersal, birds, island biogeography, extinct species

## Abstract

Seasonal migration is an underappreciated driver of animal diversification. Changes in migratory behaviour may favour the establishment of sedentary founder populations and promote speciation if there is sufficient reproductive isolation between sedentary and migratory populations. From a systematic literature review, we here quantify the role of migratory drop-off—the loss of migratory behaviour—in promoting speciation in birds on islands. We identify at least 157 independent colonization events likely initiated by migratory species that led to speciation, including 44 cases among recently extinct species. By comparing, for all islands, the proportion of island endemic species that derived from migratory drop-off with the proportion of migratory species among potential colonizers, we showed that seasonal migration has a larger effect on island endemic richness than direct dispersal. We also found that the role of migration in island colonization increases with the geographic isolation of islands. Furthermore, the success of speciation events depends in part on species biogeographic and ecological factors, here positively associated with greater range size and larger flock sizes. These results highlight the importance of shifts in migratory behaviour in the speciation process and calls for greater consideration of migratory drop-off in the biogeographic distribution of birds.

## 1. Introduction

Billions of birds travel twice a year between their breeding and wintering grounds, on journeys covering hundreds to thousands of kilometres [1]. In fact, birds undertake some of the most spectacular seasonal migrations in the animal kingdom and while seasonal migration is a globally well-studied phenomenon, little research has assessed its role in population divergence and diversification processes (see [2–4]).

Seasonal migration is here defined as regularly timed movements of organisms between breeding and non-breeding locations [5]. Most bird species, sedentary or migratory, exhibit some level of breeding site fidelity and/or philopatry and indeed, many migratory species return to their breeding grounds with exceptional precision [6]. There is thus no direct relationship between migration distance and dispersal distance (i.e. movement between birth and first breeding, or between breeding events [7]; see [8,9]). However, migratory movements may also increase the opportunities to encounter, settle and breed in, locations far from the place of birth or previous breeding [10]. In those rare cases where migratory birds attempt to breed far from their birthplace or previous breeding locations, migratory movements can effectively result in long-distance dispersal [11]. When the settlement is associated with a loss of migratory behaviour (i.e. a shift towards sedentariness, called migratory drop-off), it may result in speciation if accompanied by an interruption of gene flow between migratory and sedentary populations [12].

Phylogenetic studies in several bird clades have identified migratory drop-off as an important mechanism of speciation (e.g. [13–16]) and previous studies have underscored the significance of this phenomenon on large scales [4,12,17,18]. Despite these insights, a thorough evaluation in the specific context of island environments is lacking. Yet, island environments offer an optimal study system for delving into this mechanism, as island endemicity must result from over-water dispersal that then leads to speciation in oceanic islands (i.e. those surrounded by water since their emergence) [19].

**Figure 1.**
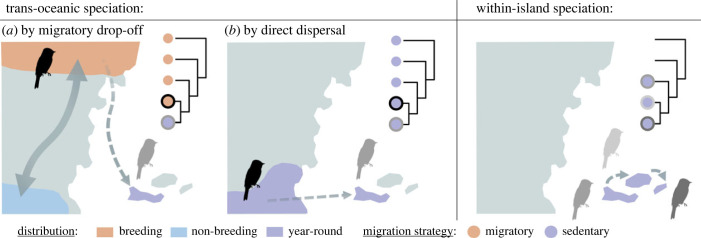
Schematic examples of the role of bird movements in the evolution of island endemic species. Trans-oceanic speciation represents a dispersal event associated with speciation, which can be induced by (*a*) migratory drop-off or (*b*) direct dispersal movements (as a result of exploratory behaviour, not related to migration). In the case of migratory drop-off, speciation results from the settlement of a migratory species on an island, accompanied by an interruption of gene flow between migratory and sedentary populations. Such events can, for example, be identified when an island endemic lineage is embedded in a migratory group of species that undertake migratory movements between breeding (pink) and non-breeding (blue) zones that pass nearby the island. The double arrow in panel (*a*) indicates the migratory routes of the closest relative, while the dotted arrow represents the one-way migratory movement that led to the colonization of the island. Conversely, speciation by direct dispersal generally concerns island endemic species embedded in a group of species restricted to a nearby continental region, mostly sedentary or which do not express (partial or strict) migratory behaviour (i.e. year-round distribution). Within-island speciation represents species derived from a unique colonization event that occupy the same island or archipelago. Silhouettes were downloaded from phylopic.org. The different grey colours indicate island endemic species whereas the black silhouette represents the closest mainland relative.

Bird immigration from continents to islands via over-water dispersal can result either from exploratory movements, which we refer to here as direct dispersal, or from migratory movements (figure 1). Migratory movements can be either seasonal or irruptive, and performed in response to environmental variation [20]. By contrast, exploratory movements are generally performed to explore the environment, regardless of any variation of the latter, and can occur before or after the breeding season, with the primary objective of finding new breeding territories [21]. Both migratory and sedentary species can perform exploratory movements, but the distances are relatively short compared to those covered during migratory movements [22]. In addition, visitation of island environments also depends on the species' degree of vagrancy, i.e. the appearance of an individual outside the normal distribution range of its species, often due to navigational errors or severe weather events, a phenomenon that is more frequent in migratory species [11,23]. Interestingly, the relative contributions of different bird movements (migration by-products versus exploration) to island colonization remain unknown.

Despite its importance in island biogeography, the process of colonization remains poorly understood [24]. Alternating phases of selection for high and low dispersal ability have long been considered a key feature in the colonization and speciation process [25–27]. In the context of trans-oceanic speciation in migratory species, variations in the use of migration routes, and the large climatic fluctuations that occurred during the Pleistocene, may also have acted as alternating phases that either favoured or prevented the arrival and settlement of migratory species on islands [28]. For example, during ice-age conditions, migratory movements were considerably reduced [29,30]. Thus, an established island population may have diverged rapidly from its original migratory population, and reproductive barriers may have continued to promote divergence once migratory movements were restored.

In this study, we assess the influence of migratory movements in promoting speciation in insular environments. We take advantage of the availability of recent comprehensive phylogenies for most bird clades (e.g. [31]) and the increase of data availability on recently extinct species (e.g. [32–34]). We first completed an exhaustive literature review, looking for evidence of trans-oceanic speciation associated with migratory drop-off on islands [19]. For each island, we estimated the proportion of endemic species resulting from migratory drop-offs, the source area on the mainland of all potential ancestors of endemic species, and the proportion of migratory species in the source area. Using these values for 40 islands, we tested if seasonal migration has a larger effect on island endemic richness than direct dispersal and therefore if seasonal migration is an important driver of speciation on islands. We then separately analysed the geographical and taxonomic variation in the importance of migratory drop-off. We tested whether the contribution of migratory drop-off changes with geographical features of islands (e.g. latitude, distance from the nearest landmass). We also tested whether traits thought to influence over-water long-distance dispersal and speciation in birds (table 1), including flight efficiency (hand-wing index), relative brain size, flock size or diet breadth, may drive the success of speciation by migratory drop-off.

**Table 1.** Definitions and predictions for the association between ecological and biogeographical traits and island colonization and speciation by migratory drop-off. We calculated mean values per family. Additional details can be found in the electronic supplementary material, Information.

traits	definition	prediction	data source
range size	the number of grid cells occupied by each species	families that occupy a larger geographic area are more likely to colonize more archipelagos	[35]
flock size	the average of mean flock size of each month of each species	probability of establishment of a sustainable population increases with the number of arriving individuals [36]	[37]
diet breadth	the number of diet categories used by each species	families with a broader diet (generalists) would be more likely to colonize islands, because of their greater ability to find resources and survive compared to dietary specialist species [38]	[39]
relative brain size	the residuals from a log-log phylogenetic generalized least square regression of absolute brain size against body size	larger relative brain sizes have been shown to reflect a disproportionate enlargement of the pallial areas and enhanced flexibility [40,41], and are suggested to be associated with island living [42]	[43,44]
hand-wing index	the Kipp's distance (the distance between the tip of the first secondary feather and the tip of the longest primary feather) corrected for wing size	hand-wing index is a surrogate for flight capacity [45]; families with a high flight capacity (i.e. strong fliers) could reach isolated archipelagos more easily than weak flyers	[46]

## 2. Material and methods

All analyses were performed in R version 4.2.2 using the packages ape 5.7–1 [47], foreign 0.8–84 [48], ggplot2 3.4.4 [49], mapplots 1.5.1 [50], phylolm 2.6.2 [51], Rphylopars 0.3.9 [52] and scales 1.2.1 [53].

### (a) Speciation process

To identify island endemic species that evolved from trans-oceanic speciation associated with migratory drop-off, we collected information on phylogenetic relationships, migratory movements and distributions for mainland and close relative species of all island endemic species. To examine the biogeographic history of lineages with minimum uncertainty, we chose to rely on comprehensive phylogenies for each clade (see below) rather than on the available global bird phylogeny [54].

First, we compiled a list of island endemic bird species, including 316 recently extinct species (i.e. from the Late Pleistocene onward; [34,35]). We were interested in most recent cases of speciation (less than 5 000 000 years ago) and our list therefore excluded families that are entirely endemic to islands (e.g. Acanthisittidae, Brachypteraciidae). We also excluded seabirds (here defined as Suliformes, Procellariiformes, Sphenisciformes, Stercorariidae and Alcidae) due to their complex movements outside of the breeding period. We excluded the few island endemic species that undertake seasonal migration (e.g. *Progne cryptoleuca*, *Progne dominicensis*). We also excluded Rallidae which, despite the presence of many endemic species on numerous islands, likely contain a disproportionally large number of unrecorded anthropogenic extinct species [55], which could bias our analyses. Note that we worked at the archipelago level (see the list in electronic supplementary material, figure S1), but because some islands are isolated and therefore not part of any archipelago, we used the term island throughout the text to avoid confusion.

Second, we identified the closest mainland relative (i.e. the continental species that shared the same most common ancestor) of each island endemic species. Mainland was here defined as continents as well as islands larger than 100 000 km^2^ (e.g. Madagascar, Cuba or New Zealand). These larger islands were considered both as ‘islands’ and ‘mainland’, acting as sources for species that have speciated on surrounding islands (i.e. mainland) but also allowing speciation of migrants from continents (i.e. island). We distinguished species derived from trans-oceanic speciation from species derived from within-island speciation (i.e. clades of multiple species derived from a unique colonization event which occupy the same island or other islands of the same archipelago). We omitted species derived from within-island speciation in the subsequent analyses since we were interested in island colonization events.

Third, we assessed whether island endemic species evolved from trans-oceanic species induced by migratory drop-off or direct dispersal movements, based on information on migratory behaviour, seasonal movements and distributions of mainland and close relatives found in reference handbooks [56,57]. Evaluating whether island endemic species have emerged via migratory drop-off or direct dispersal is not straightforward for all species and can be subject to debate. To account for the uncertainty associated with this inference, we have created four categories reflecting whether island endemic species have evolved from migratory drop-off: (1) very likely (conservative set, with clear and convincing evidence), (2) likely (liberal set, with reasonable suspicion), (3) unlikely and (4) data deficient (not sufficient evidence). A species has *very likely* evolved by migratory drop-off if the closest-relative is a strict migrant, related species (i.e. other species that share the same evolutionary history and that are grouped together on the phylogenetic tree) are strict or partial migrants, and the island is an extension of, or near, current migration routes. A species has *likely* evolved by migratory drop-off if the island is an extension of, or near, current migration routes, but the closest-relative (and related species) includes sedentary populations (i.e. is a partial migrant) and colonization by a direct dispersal event from current or past sedentary populations cannot be ruled out. A species is coded as unlikely if it is unlikely to have evolved via migratory drop-off, but is more likely to have evolved from direct dispersal movements, if the closest-relative and related species are mostly sedentary (see electronic supplementary material, Information for more details).

### (b) Seasonal migration as a driver of speciation on islands

Because island endemic species that evolved from migratory drop-off must have derived from migratory ancestors, we investigated if seasonal migration is a driver of speciation on islands.

For each island, we estimated the proportion of endemic species resulting from migratory drop-offs, the source area on the mainland of all potential ancestors of endemic species, and the proportion of migratory species in the source area. Using these values, we tested if seasonal migration has a larger effect on island endemic richness than direct dispersal by comparing the proportion of island endemic species derived by migratory drop-off to the proportion of migratory species in the source area. If, in the majority of islands, the proportion of island endemic species resulting from migratory drop-off surpasses the percentage of migratory species in the source area, it suggests that migratory species are more likely to drive the evolution of island endemic species, emphasizing the importance of drop-off speciation as an important phenomenon.

The proportion of colonization events derived by migratory drop-off was estimated from the number of island endemic species identified to have evolved from migratory drop-off (likely + very likely) against the number of species derived from direct dispersal events. The proportion of migratory species in the source area was estimated as follows. For each island, the source area was defined as the polygon encompassing the distribution of all mainland sister groups of all island endemic species. We used the breeding range maps of Birdlife International [35] gridded at a 50 × 50 km resolution to delineate the polygon and then extracted the number of migratory (partial and strict) and the number of sedentary species (i.e. the overall species richness), considering species with at least 25% of their range overlapping the polygon (migration strategies can be found in electronic supplementary material, data S1). We considered that 25% of the breeding distribution of a species represents a sufficiently significant part of its population but also tested whether different values of overlap threshold could affect the results.

Subsequently, we calculated the number of islands where the ratio of endemic species derived by migratory drop-off surpassed the proportion of migratory species in the source area. A binomial test was then employed to determine whether this count significantly deviates from the null hypothesis (H_0_ = 50%). To account for uncertainties in our categorization of migratory drop-off events, we conducted a parallel analysis only including events identified as *very likely* (with events identified as *likely* reclassified as direct dispersal events).

To ensure that we only consider trans-oceanic speciation events (i.e. exclude vicariance events), we excluded islands that have been connected to continental landmasses in the last 5 000 000 years and which have been too close (less than 100 km) to landmasses to consider that long-distance dispersal events occurred (details can be found in electronic supplementary material, figure S1).

### (c) Geographical structure of migratory drop-off

We tested for potential geographic associations of migratory drop-off using three different variables for this purpose: (1) absolute latitude, (2) distance from the nearest equivalent or larger landmass and (3) ocean identity (categorical). We expected to find a positive correlation between the proportion of island endemic species derived by migratory drop-off and the absolute latitude of islands, as the occurrence of migratory species may increase in high latitude islands because both the proportion and the number of migratory species increase with latitude. The distance from the nearest equivalent or larger landmass was defined as the distance between the island and the nearest continent or island of similar area. This variable assessed whether migratory drop-offs are more frequent on isolated islands, as we suspected that sedentary species may colonize islands located far from continents only if they manage successively to colonize islands located near each other using them as stepping stones. We used the distance values published in Valente *et al*. [58], available for most of the islands and followed their methods to estimate the distance to the nearest mainland or to the nearest equivalent or larger landmass for missing values (see details in electronic supplementary material, data S1). Finally, we used the third variable, ocean, indicating the ocean (Atlantic, Pacific, Indian; considering the Mediterranean and the Caribbean as part of the Atlantic Ocean) in which the island is located to test for potential regional effects.

We tested the relative influence of these three variables on the proportion of island endemic species derived by migratory drop-off (likely + very likely; against the number of species derived by direct dispersal events). Again, we omitted species derived from within-island speciation. We examined correlations between variables prior to the analysis and fitted a binomial model using the *glm* function with the proportion of island endemic species derived by migratory drop-off as the explanatory variable. Because differences in the number of island endemic species mostly depend on the age and surface area of the island [58], we weighted the model by the total number of endemic species in each island. We considered the same set of islands as in the previous analysis.

### (d) Biogeographic and ecological traits

We wanted to investigate whether, and if so why, some families exhibit a greater number of migratory drop-off events than others. To do so, and for migratory species only, we selected five different variables considered as relevant for influencing over-water long-distance dispersal and island speciation that we averaged by family: (1) range size, (2) flock size, (3) diet breadth, (4) relative brain size and (5) hand-wing index (for definitions and predictions table 1 and electronic supplementary material, Information). We tested for the relative influence of these biogeographic and ecological traits on the number of migratory drop-off events per migratory family using phylogenetic regression.

We modelled rate of migratory drop-off per family, calculated by multiplying the total number of drop-offs (likely + very likely) by the proportion of migratory species and divided by total evolutionary time (sum of all branch lengths). Our null expectation was that all families have the same rate of drop-off per million years per lineage and we assumed that the fraction of migratory species within each family remained constant over time (even though we do not have evidence supporting this assumption).

Using the package phylolm [51], we then fitted phylogenetic generalized least square (PGLS) models to test how our five explanatory variables (averaged per family) were associated with the rate of migratory drop-off events per family, while controlling for phylogenetic relatedness with Pagel's lambda (*λ*) [59]. We computed a family-level phylogeny, following the BirdLife International taxonomy, by pruning the maximum clade credibility (MCC) tree of Jetz *et al*. [54], modified with the backbone of Prum *et al*. ([60]; see method in [61]). All variables were centred and scaled prior to analyses to facilitate interpretation (effect sizes were obtained from regression coefficients of the model) and correlations between variables were examined prior to the analysis (all had variance inflation factors below five). Variable importance was calculated with a function where each variable was randomly permuted (1000 times) to simulate its absence in the model while keeping the number of degrees of freedom constant [62]. We considered a total of 91 families for this analysis, including 43 families with at least one migratory drop-off event identified and 48 families with no drop-off identified but including at least one migratory species.

To evaluate the importance of anthropogenic extinction and the need to consider extinct species in understanding evolutionary patterns [34], we repeated the phylogenetic regression by either excluding species extinct before 1500 CE or excluding all extinct species. To test whether these variables are specific to over-water long-distance dispersal and island speciation in migratory species, we also repeated the analysis for species having derived by direct dispersal events. Following the same method, we modelled the corrected number of direct dispersal events per family, excluding species derived from within-island speciation, considering a total of 180 families including 47 families with at least one island endemic species derived by direct dispersal. Finally, to explore whether the effects of these five variables may differ at smaller geographic scales, we repeated the analyses for the three oceans (Atlantic, Pacific, Indian) separately.

It is analytically challenging to know when to rely only on available data and when to rely on complete but potentially imprecise data coming from imputation. For the main analyses, we used only the available data for flock size (87% of species) and relative brain size (26% of species) but we ran supplementary analyses where we imputed all missing values before taking means (details in electronic supplementary material, Information). The results were very similar between these approaches (electronic supplementary material, table S1 versus S2) and the imputed results will not be discussed further.

## 3. Results

**Figure 2.**
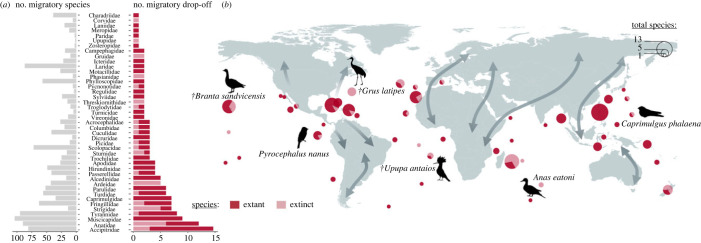
(*a*) The number of island endemic species that evolved from trans-oceanic dispersal induced by migratory drop-off identified by family, with extant and extinct (within the last 130 000 years) species depicted by different colours. (*b*) Geographical distribution of these cases of speciation induced by migratory drop-off. Silhouettes represent some examples of island endemic species (extant or extinct: †) that likely evolved from migratory drop-off. Details about species and names of the islands and archipelagos can be found in electronic supplementary material, Information. Silhouettes were downloaded from phylopic.org.

We identified 157 events of migratory drop-off that led to speciation (67 identified as very likely, 90 as likely; figure 2). Including within-island speciation, we estimated that 318 island endemic species (221 extant, 46 extinct after 1500 CE and 51 extinct before 1500 CE) likely evolved subsequently as a result these 157 unique colonization events (electronic supplementary material, figure S2).

**Figure 3.**
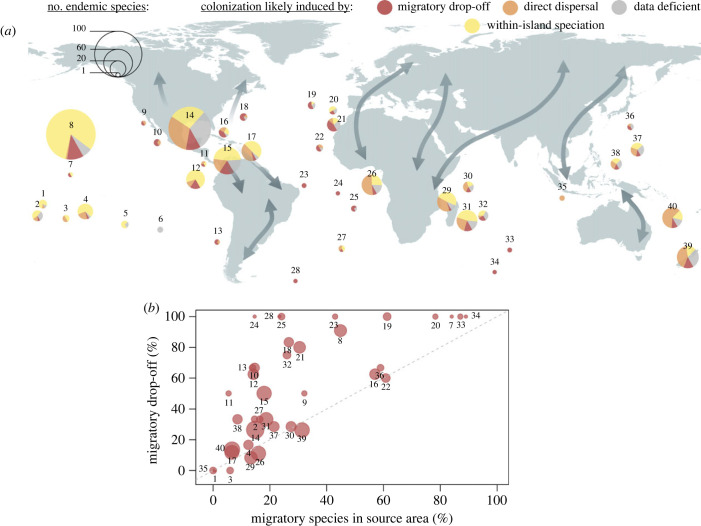
(*a*) Relative contribution of migratory drop-off in bird island endemicity. We differentiated species that evolved from trans-oceanic speciation associated with migratory drop-off (red) or with direct dispersal movements (orange). We also represented endemic species that evolved from within-island speciation (yellow) and species for which current knowledge does not allow any conclusion (data deficient; grey). The size of the pie is proportional to the number of described endemic species on the islands. Double arrows indicate main migratory flyways. (*b*) Comparison of the proportion of island endemic species that derived from migratory drop-off (likely + very likely; calculated against the number of island endemic species that derived from direct dispersal movements) with the proportion of migratory species in the source area (against the number of sedentary species). For each island, the source area was defined as a polygon delimited by the distribution of all mainland species (see Materials and methods). Eighty-two per cent of islands studied, located above this line, have a higher-than-expected number of migratory drop-off events (H_0_ = 50%; z-statistic = 3.98, *p*-value < 0.001). Names of the island or archipelago can be found in electronic supplementary material, Information.

Migratory drop-off represents a significant proportion of the colonization events for several islands. We found that very likely and likely migratory drop-offs together represent at least seven out of 14 colonization events in the Canaries (representing 11 out of 18 species), 10 out of 17 in Hawaii (representing 78 out of 95 species), three out of four in Saint Helena (representing three out of four species), and eight out of 24 in Mauritius and Reunion islands (representing 14 out of 39 species; figure 3*a*; electronic supplementary material, data S2). The proportion of island endemic species that evolved by migratory drop-off was higher than the proportion of migratory species in the source area in 31 out of 38 islands (H_0_ = 50%; confidence interval = 0.66–0.92; *p*-value < 0.001; figure 3*b*) suggesting that seasonal migration was important for speciation. We obtained very similar results when considering only the migratory drop-off events identified as very likely, and when considering different values of overlap threshold (electronic supplementary material, figure S3).

We found that the proportion of island endemic species derived from trans-oceanic speciation induced by migratory drop-off (against the number of trans-oceanic speciation induced by direct dispersal events) significantly increased with the distance of the nearest larger or equivalent land mass (table 2), suggesting that migratory drop-off is more frequent on isolated islands. These patterns were not influenced by latitude. We also found strong regional effects in the proportion of island endemic species that evolved by migratory drop-off according to the location of the islands, with drop-off events occurring more often in the Atlantic Ocean (table 2).

**Table 2.** Results of the binomial regression modelling the effect of geographical variables on the proportion of island endemic species derived per migratory drop-off. The distance to equivalent island represents the distance of the nearest larger or equivalent land mass and assesses whether migratory drop-offs are more frequent on isolated islands. s.e. is the standard-error and significant *p*-values are denoted with asterisks; *p* < 0.05 (*), *p* < 0.01 (**) and *p* < 0.001 (***).

variable	estimate	s.e.	*z*-value	*p*-value
Atlantic Ocean	−0.8	0.3	−2.8	0.005**
Indian Ocean	−1.5	0.4	−3.9	<0.001***
Pacific Ocean	−1.6	0.4	−4.2	<0.001***
latitude	1.0	0.7	1.4	0.156
distance to equivalent island	2.4	0.7	3.5	<0.001***

**Figure 4.**
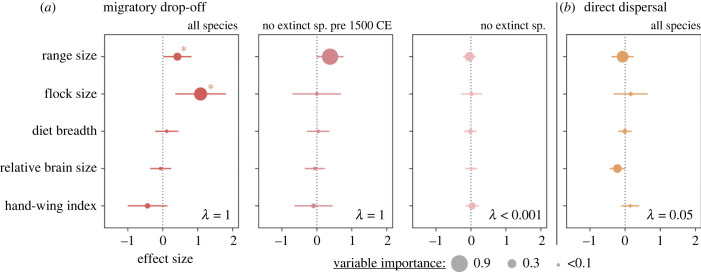
Effects of biogeographic and ecological traits on trans-oceanic speciation by migratory drop-off (*a*) or direct dispersal (*b*), based on differences found between families. We tested different models considering all extant and extinct species, only species alive to at least 1500 CE and only extant species. Effect size and relative importance were estimated from phylogenetic generalized least squares (PGLS) regression models. Lambda values are indicated in the bottom right of each box. Asterisks denote a significant effect size at *p*-value = 0.05 (details can be found in electronic supplementary material, table S1).

Biogeographic and ecological characteristics can facilitate speciation by migratory drop-off and may explain why some migratory families were more successful in speciating in island environments than others. Our models identified a positive effect of range size and flock size on the corrected number of migratory drop-off events identified per family (figure 4*a*; electronic supplementary material, table S1): migratory families travelling in large flocks and/or species that occupy large geographic areas are more likely to colonize an island to speciate (respective *p*-values = 0.004 and 0.044). By contrast, we did not find any effect of diet breadth, relative brain size or hand-wing index. By conducting the same analysis for the three oceans separately, we found that the effect of flock size was predominant for the islands of the Atlantic, while range size has a greater effect in the Pacific and Indian oceans (electronic supplementary material, figure S4). We also found a negative effect of the hand-wing index in the Atlantic, suggesting that strong fliers in this area might have a negative effect on speciation on islands (electronic supplementary material, figure S4). Analyses that excluded either species extinct before 1500 CE or all extinct species failed to recover any significant effects (figure 4*a*) and the strong phylogenetic signal that we found when all species were included (*λ* = 1) disappeared when all extinct species were excluded (*λ* < 0.001; electronic supplementary material, table S1). Note that exploratory analyses found that the number of migratory drop-off events per family scaled linearly with the proportion of migratory species (correlation coefficient = 0.77) and with total evolutionary time (correlation coefficient = 0.58), suggesting that the approach is justified (more details can be found in electronic supplementary material, Information).

## 4. Discussion

### (a) Seasonal migration and island speciation

Here, we identify endemic species on most of the world's islands that evolved from trans-oceanic speciation induced by migratory drop-off of a likely migratory ancestor. In most islands, we found a higher proportion of island endemic species that evolved from migratory ancestors than the proportion of migratory species among potential colonizers, suggesting that seasonal migration has a larger effect on island endemic richness than direct dispersal and hence plays an important role in the immigration process on island biogeography. Our results indicate a positive influence of migratory behaviour on speciation rate by promoting long-distance dispersals on remote islands where divergence can rapidly occur, and hence corroborate the main results of Rolland *et al.* [4] (see also [12]). Rolland *et al.* [4] indeed found that migratory species exhibit a higher net diversification rate than sedentary species and suggest that migratory drop-off has facilitated diversification on a global scale.

While we have identified island endemic species that likely evolved from migratory drop-off on most islands, the relative importance of this phenomenon compared to direct dispersal events appears to be variable across islands. As examples, we identified few migratory drop-off events in proportion to direct dispersal events (or species evolved via within-island speciation) in the southern Pacific Ocean, while migratory drop-off events constitute most of the colonization events for the mid-Atlantic islands (e.g. Azores, St Helena). A taxonomic and biogeographic effect can be expected due to the peculiar distribution of certain families which show numerous migratory drop-off events. The Fringillidae, for example, are found on almost every continent but are particularly diverse in the Holarctic regions, with consequently more frequent examples of island colonization in the Northern Hemisphere (e.g. Canary Islands). Conversely, several families composed almost exclusively of sedentary species, such as the Meliphagidae, Petroicidae and Rhipiduridae, have diversified in Oceania, where they represent numerous direct dispersal events for several islands. In addition, we found that the proportion of migratory drop-off events increases on isolated islands, suggesting that migratory species are disproportionally more likely to occur, settle and diverge on islands isolated from the continents, and from other islands of at least similar size, than sedentary species. This result also suggests that isolated islands are less accessible for sedentary species, which can potentially only reach islands close to the mainland or close to each other. This pattern is consistent with the idea of the stepping-stone model of island colonization, mostly proposed for sedentary lineages, where the first settlers come from the nearest mainland and then follow a chronological sequence of colonization of the nearest island [63].

Our results also suggest that the proportion of drop-offs increases for islands located within or close to major migratory flyways. We found many drop-offs for the Caribbean islands or the Canary Islands, but few drop-offs in the many islands of the Pacific Ocean. Even though the Caribbean and the Canary Islands are close to the continental masses (or to other islands of similar size) where gene flow may be favoured and thus inhibit the speciation process, their proximity to major migratory flyways probably compensates for this. Indeed, their geographical location makes these islands more likely to be visited by migratory species, which may be trapped and/or encounter environmental conditions that influence their decision to migrate and promote migratory drop-off [13].

The location of insular endemic species with respect to the geographical origin of migratory lineages corroborates the hypothesis that migratory species have a high propensity for vagrancy, and therefore to occur on isolated islands where they may act as a propagule in the colonization phase [64]. Several cases of migratory drop-off indicate that migratory birds do not necessarily originate from the nearest land. In Hawaii for example, although the archipelago is almost twice as close to America as it is to Asia, several island endemic lineages derive from Asian migratory species (e.g. honeycreepers: [65]; crows: [66]). The same is true for several island endemic species of the Mascarene archipelago which derived from migratory lineages that ancestrally migrated within Asia or between Asia and East Africa [67,68]. Migratory individuals departing alone on their first migrations can occasionally misinterpret their migratory programme and take erroneous orientations (e.g. mirror-image, reverse migration [69]). In addition, external meteorological factors (e.g. storms) can displace migratory birds off their usual migration routes [70]. Nevertheless, Lees & Gilroy [71], who focused on the contemporary distribution and occurrence patterns of species (thus excluding island endemic species and past colonization events), failed to find an association between colonization of oceanic islands and vagrancy in their analysis. Their study nevertheless attests that island colonization by vagrant individuals is a well-established phenomenon, more frequent in migratory species.

### (b) Ecological drivers of migratory drop-off

Our results showed that some families are more likely to perform trans-oceanic speciation through migratory drop-off than others, depending notably on the average range size and flock size of species within the families.

The positive relationship between range size and speciation would suggest that part of the variation in migratory drop-off may be driven by variation in population size. All else being equal, species with larger ranges should have more individuals and likely also more individuals outside the regular ranges (i.e. vagrants). Moreover, as hypothesized by Lees & Gilroy [71], the positive relationship could suggest that families occupying large global ranges are likely to have increased opportunities for visiting islands, as their regular range more likely encompasses or lies relatively close to multiple islands. The families that record many migratory drop-off events are logically families with a widespread distribution and/or composed of species with large ranges. Hence, a large number of migratory Muscicapidae exhibit large breeding ranges that encompass a large part of the Eurasian continent [72].

Migratory drop-off occurred particularly in families migrating in large flocks, suggesting, that the number of arriving individuals influences the probability of establishment of a sustainable population [36]. It is expected that a large group of individuals, rather than several colonization events of small groups of individuals, will allow for more successful colonization and divergence. In this perspective, we found that Fringillidae, Turdidae or Anatidae which mostly migrate in large flocks largely contributed to migratory drop-offs events. As an example, the hypothesis of a relatively large group of original colonizers has been recently suggested for the colonization of the Azores by the ancestor of the common chaffinch *Fringilla coelebs* and the Azores chaffinch *Fringilla moreletti* [73], which is consistent with the flocking behaviour of the common chaffinch. In addition, species migrating in large flocks are partly species migrating by following conspecifics (e.g. Anatidae and Gruidae [74]). Such species can more easily be trapped and become sedentary if they reach an unknown destination or if non-experienced individuals become separated from experienced individuals (e.g. [48]). Even if there is very little information available about the time it takes for a large group of individuals to speciate when colonizing a new region, it has been shown that a reproductively isolated lineage can rapidly emerged from a single individual in a vagrancy-like context [75].

Despite strong biological expectation, we found little evidence for a relationship at the family-level between speciation induced by migratory drop-off and other plausible traits directly linked to migration capacity and establishment success. Our analysis did not support an association between migratory drop-off and diet breadth, relative brain size and hand-wing index. We hypothesized that dietary generalists should have higher chances of finding suitable resources and may cope better with seasonally available resources than dietary specialists upon establishment on islands [76], which could also influence the decision to settle [77,78]. It is possible that the categorization of the diet data used was too coarse to detect an effect. Regarding relative brain size, previous work found that larger brain sizes were not associated with the propensity of island colonization, but instead seem to be favoured after the colonization event [42]. Nevertheless, further research conducted at smaller taxonomic scales might clarify the generality of these findings. A further aspect to consider is the unimodal relationship between diversification rate and dispersal ability found in several studies, where efficacy of barriers to gene flow decreases as the dispersal capacity of families increases [45,79]. A species that has colonized an island but still reaches it frequently, because of high dispersal capacity and/or frequent departure from its migratory route, could potentially maintain a sufficient gene flow to prevent divergence of populations and evolution of new species, as the case for example in Eurasian woodcock *Scolopax rusticola* and common wood pigeon *Columba palumbus* in the Canaries and Azores [80,81]. In fact, migratory species that frequently disperse to insular environments would likely have frequent short-term populations on islands during suitable periods, and their local adaptation could only depend on the change of migration routes, linked to fitness consequences [82] or climatic variations [29].

Finally, our results highlight the importance of integrating extinct species into analyses when studying global evolutionary and ecological patterns. When excluding extinct species in the comparative analyses, we missed the significant role of ecological traits (range size and flock size) as drivers of migratory drop-off colonization success and lost the phylogenetic signal. Biases in trait effects have frequently been reported previously [34,83], but the loss of phylogenetic signal when extinct species are excluded is a somewhat unexpected result. These results stress the importance of analysing the full natural diversity rather than the diminished current diversity whenever possible in macro-scale analyses. If this study stresses the importance of shifts in migratory behaviour in speciation process in island environments, the number of events identified is also likely minimized given the large number of species that have gone extinct recently without leaving any trace in the fossil record [84].

### (c) Perspectives and conclusion

Several studies have shown that migratory drop-off is an important mechanism of speciation and migratory behaviour is known to be a labile trait on evolutionary timescales (e.g. [4,17,57]). Interestingly, previous work reported that losses of migratory behaviour were more frequent than gains [4]. However, the alternative scenario of a gain in migration following a dispersal event is also theoretically plausible and might be difficult to detect from phylogeny. In the island context, such a scenario seems unlikely as the number of island migrants is limited to a few species (e.g. Cuban martin *Progne cryptoleuca*, Caribbean martin *Progne dominicensis* in the Caribbean) but invites further research to understand the context in which these species evolved.

Many movements, whether migratory or exploratory (here qualified as direct dispersal, and which can be performed by sedentary or migratory species), can lead to island colonization and result, by definition, in a dispersal movement once breeding has occurred [7]. Interestingly, it has long been suspected that migratory behaviour has a strong effect on dispersal distance [85] but recent studies suggest instead that migration and dispersal may be decoupled [9,86]. In fact, most migratory species show a trend of increasing dispersal distances with increasing flight efficiency that is very similar to the one shown by sedentary species [8]. What makes migratory species truly unique is the long-distance movements they make twice a year, which allow them to cross a multitude of localities and habitats, and which can also take them more frequently to isolated localities, like islands. In most cases identified here, the colonization of islands located thousands of kilometres away from the regular breeding grounds of the species is more likely to result as by-products of migratory movements than from exploratory movements [22]. However, the highly dispersive character of some clades that do not undertake seasonal migration movements but can perform long over-water flights (e.g. *Caloenas* sp.; [87]) still invites further research of the links between dispersal and migration, especially in these so-called highly dispersive species (see [88]). It would thus be interesting to test, at finer taxonomic scales, how differences in traits between sedentary and migratory species can influence colonization success.

## Data Availability

Data supporting the results are archived in Dryad (https://doi.org/10.5061/dryad.3tx95x6pk) [89]. Note that bird range maps and diet data are publicly available, respectively in www.birdlife.org and [39]. The repository contains a table containing all species' migration characteristics, range size, inferred flock size, and relative brain size data (Dryad, Supplementary Data S1), as well as a table with details regarding the evolutionary history and migratory behaviour of island endemic and related species (Dryad, Supplementary Data S2). Supplementary material is available online [90].
